# Analysis of pathological characteristics and nursing intervention of patients with gastric polyps based on image stitching algorithm and endoscopy

**DOI:** 10.12669/pjms.37.6-WIT.4854

**Published:** 2021

**Authors:** Linzhen Zhu, Linlin Zhu, Weihua Yu

**Affiliations:** 1 Linzhen Zhu, Deputy Director of the nurse. Endoscopy Center, The Fourth Affiliated Hospital of Zhejiang University School of Medicine, Yiwu City, 322000, Zhejiang Province, China; 2 Linlin Zhu, Deputy Director of the nurse. Beiyuan Street Community Health Service Centre, Yiwu City, 322000, Zhejiang Province, China; 3 Weihua Yu, Attending Physician. Department of Digestive Medicine, The Fourth Affiliated Hospital of Zhejiang University School of Medicine, Yiwu City, 322000, Zhejiang Province, China

**Keywords:** Pathological characteristics, Nursing, Gastric polyps, Image stitching algorithm, Endoscopy

## Abstract

**Objectives:**

The paper uses image stitching algorithm to understand the clinical and pathological characteristics of gastric polyps under gastroscope, and provides objective basis for the clinical diagnosis and treatment of gastric polyps and nursing intervention.

**Methods:**

The endoscopic, pathological data and surgical conditions of 111 patients with gastric polyps detected in the hospital from January 2017 to August 2019 were retrospectively analyzed.

**Results:**

The elderly patients (≥60 years old) in this group were those with high incidence of gastric polyps (56.8%); 80 patients with single polyps (72.1%), 31 patients with multiple polyps (27.9%); polyps were mainly located in the stomach (53.2%); polyps diameter ≤0.5cm are more common (69.4%); polyps are mainly hyperplastic polyps (40.5%) and inflammatory polyps in 37 cases (33.3%). Polyps were removed by biopsy forceps in 30 cases, endoscopic submucosal injection of 0.9% NaCl solution combined with high-frequency electrosurgical removal of 54 cases, endoscopic mucosal resection (EMR) in 6 cases, and endoscopic submucosal dissection (ESD) in treatment of the 4 cases, the remaining 17 cases were treated with surgery, and 12 patients were followed up, 2 of whom relapsed.

**Conclusion:**

Gastric polyps are small in diameter and mostly single; polyps are mainly located in the stomach body, mainly hyperplastic polyps; treatment methods are mostly endoscopic resection, and there is a possibility of recurrence after polypectomy, and follow-up should be strengthened. Full preparation before the operation, close cooperation during the operation, and careful postoperative care are important links to ensure the safety of the operation and reduce complications such as upper gastrointestinal bleeding.

## INTRODUCTION

Gastric polyps refer to neoplasms that originate from the mucosal epithelium, bulge on the surface of the mucosa, and protrude into the cavity.^1^,^2^ The vast majority of gastric polyps are benign and not life-threatening, but some polyps possibly become cancerous, and therefore require active treatment.^3^-^5^

This article retrospectively analyzed the endoscopy, pathological data and operation of 111 patients with gastric polyps detected in our hospital in the past three years to understand the clinical and pathological characteristics of gastric polyps under gastroscopy. Provide an objective basis for clinical diagnosis and treatment.

## METHODS

We included 111 inpatients with gastric polyps detected in our hospital from January 2017 to August 2019. The diagnosis was confirmed by postoperative pathological examination.

###  Exclusion criteria

1) outpatients; 2) patients who were not diagnosed with gastric polyps under endoscopy; 3) patients who were not treated; 4) patients who lacked pathological diagnosis of polyps.^6^-^8^

The patient’s education should be done well so that he understands the necessity of removing polyps and the surgical procedure. Find out the clotting time, and explain to the patients and their families the possible complications and treatment measures during and after surgery, so that the patients have full mental preparation.^9^-^11^ After the operation, the patient should be transported to the ward with a flat car or wheelchair and given symptomatic treatment such as fluid support. Local complications of high-frequency electrical resection polyp or electrocautery polyp include perforation, bleeding, and mucosal burns, among which bleeding is the most common. After pulling out the gastroscope, do appropriate stay observation and spray wine under the microscope to stop bleeding. Most patients can be prevented by intravenous drip or intramuscular injection of hemostatic drugs after returning to the ward^12^-^14^

### Statistical Analysis

Data SPSS17.0 software was used to analyze the age, size, location and pathological type of polyps.^15^ The traditional SIFT feature matching algorithm can be classified as the problem of finding feature points on different scale spaces. Because of its good invariance, it is widely used in image feature extraction and matching, but the extracted feature points often contain a large number of mismatched feature point pairs, so it is improved and a pre-test strategy. The specific implementation steps of the traditional SIFT algorithm are that the key points are some local extreme points with orientation information detected in images of different scale spaces that are very prominent and will not disappear due to changes in lighting conditions. The Gaussian kernel is the only transform kernel that can produce a multi-scale space. The scale space L(x, y, σ) of a function is defined as the input image I(x, y) (representing the pixel value of the image at position (x, y) ) Convolution with a variable-scale two-dimensional Gaussian function G (x, y, σ) (σ is the standard deviation of Gaussian normal distribution, called scale space factor).


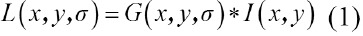



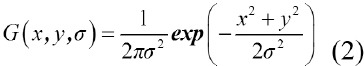


To more effectively detect stable feature points in the scale space and simplify the operation, the Gaussian difference function D (x, y, σ) (k is the scale of the scale factor of two adjacent images):


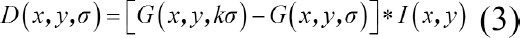


Define the following features to determine whether the key point is an exact matching feature point pair.


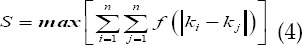


In the formula: ki, kj represent the slope of the i-th and j-th straight lines, respectively.


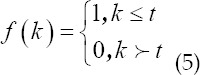


In the formula: t is the neighborhood threshold (this article takes 0.02 according to the empirical value). The specific pre-inspection strategy implementation steps are as follows: Step-1: Transform the original image and the target image into the same coordinate system, calculate the slope value of all straight lines in the coarse matching data set; Step 2: Calculate the value from feature 1 and feature 2 in a loop, and remember Under this value, the point pair set in the neighborhood is calculated as the pre-selected points to be matched, and the point pair set outside the neighborhood is deleted; Step 3: Loop through the entire coarse matching feature point set in this loop to obtain the initial screening Match feature point set. If the two adjacent feature points m, n in the original image match the feature points m’, n’ in the target image, respectively, then the parallax gradient should be less than 2.


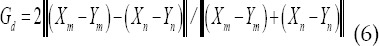


In the formula: (Xm, Ym) and (Xn, Yn) are the image coordinate vectors corresponding to the feature points;

**Table-I:** Age distribution of patients with gastric polyps and pathological types of polyps (n).

*Age*	*Hyperplastic polyp*	*Inflammatory polyp*	*Gastric polyps*	*Metaplastic polyp*	*Hamartoma*	*Adenomatous polyp*	*Gastric cancer*
≤44 years old	10	4	0	1	0	0	2
44~60 years old	9	14	2	1	1	0	4
≥60 years old	26	19	4	0	0	4	10

**Table-II:** Stomach polyps’ size and polyp pathology type (n).

*Maximum diameter (cm)*	*Hyperplastic polyp*	*Inflammatory polyp*	*Gastric polyp*	*Metaplastic polyp*	*Hamartoma polyp*	*Adenomatous*	*Gastric cancer*
≤0.5	36	33	5	1	0	2	0
0.5~2.0	6	4	1	0	0	0	1
≥2.0	3	0	0	1	1	2	15

## RESULTS

Of the 111 patients with gastric polyps included, 52 were male and 59 were female, aged 27-84 years, with an average of (60.2±12.9) years; 63 elderly patients ≥60 years old (56.8%), 17 patients ≤44 years old (15.3%), 31 cases (27.9%) were 44 to 60 years old.

. A total of 165 polyps were detected in 111 patients with gastric polyps, 80 (72.1%) patients were single polyps, 31 polyps (27.9%), including 20 polyps and six polyps. There were two cases with four polyps and three cases with five or more polyps. Polyps were mainly located in the stomach body in 58 cases (52.2%), followed by gastric antrum in 28 cases (25.2%), gastric fundus in 16 cases (14.4%), gastric cardia in five cases (4.5%), and gastric horn in two cases (1.8%). two cases (1.8%). There were 77 cases (69.4%) with polyp diameter ≤0.5cm, 12 cases (10.8%) with 0.5~2.0cm, and 22 cases (19.8%) with ≥2.0cm.

There were 111 patients with gastric polyps, 91 were non-tumor polyps, including 45 hyperplastic polyps (40.5%), 37 inflammatory polyps (33.3%), 6 gastric fundus polyps (5.4%), and metaplastic polyps. There were one case (1.8%), one case of hamartoma (0.9%); 20 cases of neoplastic polyps, including four cases of adenomatous polyps (3.6%) and 16 cases of adenocarcinoma (14.4%).

. In this group of patients, 30 cases of polyps were removed by biopsy forceps, 54 cases were removed by endoscopic submucosal injection of 0.9% NaCl solution combined with high-frequency electrosurgical removal, six cases were treated by endoscopic mucosal resection technology, and four cases were treated by endoscopic submucosal dissection. More than 17 cases underwent surgical treatment. In this group, only one patient underwent endoscopic submucosal injection of 0.9% NaCl solution combined with high-frequency electrosurgical treatment. Hemorrhage occurred during the operation, and hemostasis was given by endoscopic argon ion coagulation (APC). All patients were given acid suppression therapy for one week after operation, and no obvious discomfort was found. Twelve patients were followed up for a period of 3 to 24 months. Two patients relapsed, of which 1 patient had a single polyp recurrence and 1 patient had a multiple polyp recurrence.

**Table-III:** Locations of gastric polyps and pathological types of polyps (n).

*Part*	*Hyperplastic polyp*	*Inflammatory polyp*	*Gastric polyp*	*Metaplastic polyp*	*Hamartoma polyp*	*Adenomatous polyp*	*Gastric cancer*
Stomach body	21	17	4	1	1	4	10
Antrum	12	13	0	1	0	0	2
Fundus	9	5	2	0	0	0	0
Cardia	0	1	0	0	0	0	4
Stomach angle	2	0	0	0	0	0	0
Multi-site	1	1	0	0	0	0	0

## DISCUSSION

Gastric polyp is a common gastrointestinal disease in clinical practice, with very few clinical symptoms. Occasionally, it manifests as upper abdominal pain and discomfort and gastric bleeding. Early attention to gastric polyps can effectively prevent cancer, especially adenomatous polyps which have a very high canceration rate. Ginsberg^16^ have shown that, regardless of the size of polyps, if they are not treated in time, there will be a risk of deterioration. Timely and effective prognostic follow-up is conducive to the control of gastric cancer. In this study, all patients were followed up for one week and found to have discomfort. The endoscopic examination of gastric polyps has the advantages of less trauma, safety and reliability, fast recovery, and simple operation. With the continuous development of the endoscopic detection technology, it is widely used in clinical practice. Cui^17^ performed endoscopic mucosal resection on 70 patients, and it was radically removed in 68 of them by one surgery, and it was removed in remaining two cases in stages. There was no hemorrhoea or perforation during the operation, which also confirmed the safety and reliability of endoscopic mucosal resection in the treatment of gastric polyps. In this study, six patients were treated with endoscopic mucosal resection, and no obvious bleeding was observed. In one case treated by high-frequency electrocautery after endoscopic mucosal injection of 0.9% NaCl solution, hemorrhage occurred during the operation and was stopped by APC. This may be related to the use of high-frequency electrocautery.

Image stitching algorithms are widely used in the fields of computer and medical image processing. The process of image splicing mainly includes image preprocessing, image registration, model building, unifying coordinates, and other steps. The image registration technology is fast and accurate. Many scholars have conducted in-depth research on image processing, image preprocessing, and image fusion in order to obtain more natural image effects. However, image noise, artifacts, and distortion still exist. Mamone^18^ applied the image stitching algorithm in aortic valve replacement surgery, which provided a wider field of vision, playing an important role in the development of the endoscopic field of vision system. To use image stitching algorithm to merge the planar views of the surgical site into a single stitched image can obtain better aortic guidance and orientation measurement capabilities. In this study, the image stitching algorithm was applied in the endoscopic examination of gastric polyps, and it increased image clarity, removed image noise, so that the doctor can accurately distinguish the type of polyp through the image, and analyze its pathological characteristics, thereby choosing a reasonable treatment plan according to the type of gastric polyps. Imaging results showed that, there were 31 cases of multiple polyps, 80 cases of single polyp, and 69.4% of polyps with a diameter of ≤0.5cm. The image stitching algorithm is very effective in examining gastric polyps and is worthy of clinical promotion.

## CONCLUSION

The results of this group of data suggest that gastric polyps are more common in single cases. The most common growth site is the stomach body, followed by the gastric antrum. The elderly ≥60 years old are the people with high incidence of gastric polyps. The histopathological classification is mainly hyperplastic polyps and inflammatory polyps. Endoscopic resection is the preferred method for treating gastric polyps, mainly endoscopic polyp resection and endoscopic forceps removal, etc. Complications are rare. There is a possibility of recurrence after polypectomy, and follow-up should be strengthened.

### Authors Contribution:

**LZ:** Conceived the study, literature review, data analysis drafting the manuscript and also the responsible and accountable for the accuracy or integrity of the work.

**LZ:** Takes the responsibility and is accountable for all aspects of the work in ensuring that questions related to the accuracy or integrity of any part of the work are appropriately investigated and resolved.

**WY:** Helped in design, data collection, drafting the paper & critical revision.
